# Metagenomic sequencing complements routine diagnostics in identifying viral pathogens in lung transplant recipients with unknown etiology of respiratory infection

**DOI:** 10.1371/journal.pone.0177340

**Published:** 2017-05-23

**Authors:** Dagmara W. Lewandowska, Peter W. Schreiber, Macé M. Schuurmans, Bettina Ruehe, Osvaldo Zagordi, Cornelia Bayard, Michael Greiner, Fabienne D. Geissberger, Riccarda Capaul, Andrea Zbinden, Jürg Böni, Christian Benden, Nicolas J. Mueller, Alexandra Trkola, Michael Huber

**Affiliations:** 1 Institute of Medical Virology, University of Zurich, Zurich, Switzerland; 2 Division of Infectious Diseases and Hospital Epidemiology, University Hospital Zurich, Zurich, Switzerland; 3 Division of Pulmonary Medicine, University Hospital Zurich, Zurich, Switzerland; Kliniken der Stadt Köln gGmbH, GERMANY

## Abstract

**Background:**

Lung transplant patients are a vulnerable group of immunosuppressed patients that are prone to frequent respiratory infections. We studied 60 episodes of respiratory symptoms in 71 lung transplant patients. Almost half of these episodes were of unknown infectious etiology despite extensive routine diagnostic testing.

**Methods:**

We re-analyzed respiratory samples of all episodes with undetermined etiology in order to detect potential viral pathogens missed/not accounted for in routine diagnostics. Respiratory samples were enriched for viruses by filtration and nuclease digestion, whole nucleic acids extracted and randomly amplified before high throughput metagenomic virus sequencing. Viruses were identified by a bioinformatic pipeline and confirmed and quantified using specific real-time PCR.

**Results:**

In completion of routine diagnostics, we identified and confirmed a viral etiology of infection by our metagenomic approach in four patients (three Rhinovirus A, one Rhinovirus B infection) despite initial negative results in specific multiplex PCR. Notably, the majority of samples were also positive for Torque teno virus (TTV) and Human Herpesvirus 7 (HHV-7). While TTV viral loads increased with immunosuppression in both throat swabs and blood samples, HHV-7 remained at low levels throughout the observation period and was restricted to the respiratory tract.

**Conclusion:**

This study highlights the potential of metagenomic sequencing for virus diagnostics in cases with previously unknown etiology of infection and in complex diagnostic situations such as in immunocompromised hosts.

## Introduction

Post-transplant immunosuppression significantly increases the risk of viral infections in lung transplant recipients [1–3]. Infections commonly considered to cause mild or self-limiting disease in immunocompetent hosts, can lead to unusual presentations with increased severity, mortality or duration [4–6] and numerous community-acquired respiratory viral infections are implicated in triggering rejection episodes [7]. As a consequence, lung transplant recipients are frequently tested for a range of common respiratory viruses, bacteria and fungi [8–10]. Despite this rigorous testing, a considerable number of episodes of respiratory symptoms remain unresolved [11]. Successful diagnosis would, however, be important for several reasons. Specific treatment is available for most bacterial and fungal pathogens and some viruses. A rapid confirmation reduces unnecessary diagnostic steps and allows to set effective measures to restrict nosocomial transmission. Excluding potential transmissible pathogens as source of the etiology is therefore equally important as their confirmation.

Most transplant centers employ a tight monitoring for clinical signs of respiratory infection in lung transplant patients over prolonged periods after transplantation [12]. The panel of routinely assessed respiratory viruses has been continuously expanded in recent years. Currently, many programs use multiplex PCR analysis targeting up to 20 different viruses, however, still not all viruses potentially relevant to the lung transplant setting can be included. Considering the high incidence of airway inflammation following lung transplantation, close monitoring of the virome may be advantageous in linking distinct pathogen signatures with specific outcomes, such as allograft dysfunction.

The rise of high-throughput sequencing, also called Next Generation Sequencing, combined with virus-sequence independent amplification by random PCR allows for unbiased detection of virtually any pathogen present in a given sample [13–15]. This methodology may overcome one of the great limitations of routine virus diagnostics, which restricts the number of detectable pathogens to the ones included in the assay. Here, we established and used this approach for re-analyzing respiratory samples from lung transplant patients presenting with symptoms suggestive of an airway infection for which no viral or microbial etiology was found by extensive routine diagnostic methods with the aim to resolve a viral etiology of infection using metagenomic sequencing.

## Material and methods

### Ethical statement

Samples were obtained from lung transplant patients and healthy controls within the Viral Metagenome Study of the Clinical Research Priority Program ‘Viral Infectious Diseases’ of the University of Zurich. The Ethics Committee of the Canton of Zurich approved the study and written informed consent was obtained from all participants. None of the transplant donors were from a vulnerable population and all donors or next of kin provided written informed consent that was freely given.

### Transplant cohort

The study was performed at the University Hospital Zurich, Switzerland, which is a 900 bed tertiary care teaching hospital covering all medical specialties except pediatrics and orthopedics. Since May 2013 patients listed for lung transplantation and patients with prior lung transplant have been asked for participation in the current study. Inclusion criteria were age of at least 18 years and written informed consent. The willingness to participate in the study was notable, 83.8% of asked individuals consented. Lung transplant recipients were either included at the time of transplantation (baseline enrollment) or at the time of presentation with symptoms suggestive of an airway infection (indication enrollment). Participants enrolled at baseline had planned study visits at the time of transplantation, 4–6 weeks and 1 year thereafter. Drop out rate was low with 4.1%. Additional visits were scheduled, if a patient developed symptoms suggesting an airway infection. After every indication enrollment or symptom visit a follow-up visit was scheduled. At each visit, throat swabs, plasma, urine and stool samples were obtained and patients were evaluated for signs of infection and relevant transplant outcome events. An airway infection was considered if cough and/or dyspnea and/or a decrease in forced expiratory volume in 1 second (FEV1) were present (200 ml or 10% decrease [12, 16]). Fever alone was not considered sufficient to define an airway infection.

### Healthy controls

Healthy donors were recruited at the University of Zurich and questioned for co-morbidities. Throat swabs were obtained as for the lung transplant recipients.

### Routine immunosuppression

Induction immunosuppression consisted of basiliximab (20 mg on day of surgery and post-operative day 4) and methylprednisolone (1 g given during operative procedure, 125 mg on postoperative day 1 and 2). Maintenance immunosuppression included mycophenolate mofetil, prednisone and a calcineurin inhibitor, primarily cyclosporine [17].

### Antimicrobial prophylaxis

Lung transplant recipients received antibacterial and antifungal prophylaxis (sulfamethoxazole/trimethoprim, itraconazole and aerosolized amphotericin B). Anti-viral prophylaxis was administered based on CMV-risk constellation depending on serology of donor and recipients. In intermediate (donor CMV negative or positive/recipient CMV positive) or high risk (donor positive/recipient negative) constellations valganciclovir was used, whereas in low risk constellation (donor CMV negative/recipient CMV negative) valaciclovir as prophylaxis against HSV, VZV was administered [17]. All prophylactic regimens were usually prescribed lifelong.

### Routine viral and microbial testing

Routine workup included throat swabs or broncho-alveolar lavage (BAL) for viral and microbial analysis. Respiratory viruses were tested using the respiratory pathogens 21 panel (Fast-track Diagnostics, Junglinster, Luxembourg), which detects 18 different respiratory virus types (Influenza A, Influenza A H1N1 pdm 09, and Influenza B viruses; Coronaviruses NL63, 229E, OC43 and HKU1; Parainfluenzavirus 1, 2, 3 and 4; Human Metapneumovirus A/B, Rhinovirus, Respiratory Syncytial viruses A/B, Adenovirus, Enterovirus, Parechovirus and Bocavirus) and *Mycoplasma pneumoniae*. CMV and EBV were tested in blood samples using specific PCR [18, 19]. Routine cultures for bacteria and fungi were performed for all samples sent to the microbiology laboratory.

### Metagenomic sequencing and bioinformatic analysis

Throat swab samples were collected in 3 ml virus transport medium (HEPES buffer pH 7.4, 10% FCS, antibiotics); BAL samples were collected native. To enrich for free virus particles, samples were centrifuged at 1500 rpm for 5 min and the supernatant filtered through a 0.45 μm filter. Total nucleic acids were extracted from 1000 μl of filtrate with a NucliSENS EasyMAG (BioMérieux, Craponne, France) in an elution volume of 25 μl. RNA and DNA were amplified in two separate workflows. For RNA amplification, a random primer with an anchor sequence (ATCGTCGTCGTAGGCTGCTCNNNNNNNN, 5 μM) [20–22] was used for reverse transcription (SuperScript III, Invitrogen/Thermo Fisher, Waltham, MA) of 5 μl eluate, performed at 42°C for 60 min. For the DNA workflow, 5 μl of the eluate was incubated with the same random primer as in the RNA workflow at 94°C for 2 min and cooled down to 10°C for 10 min. One round of second-strand synthesis (Large Klenow Fragment, New England Biolabs, Ipswich, MA) with 5 U/μl enzyme per reaction was performed in both workflows at 37°C for 30 min, followed by inactivation at 75°C for 20 min. In both workflows, nucleic acids were further amplified with AmpliTaq Gold (Applied Biosystems/Thermo Fisher) using only the anchor primer (1 μM) and the following temperature protocol: 94°C for 15 min; 40 cycles of 94°C for 30 s, 40°C for 30 s, 50°C for 30 s, 72°C for 1 min; 72°C for 5 min. Finally, both workflows were pooled in equal amounts for library construction with NexteraXT (Illumina, San Diego, CA) prior to sequencing for 150 bp on an Illumina MiSeq with version 3 reagents and the “FASTQ only” workflow. Samples were demultiplexed using MiSeq Reporter v2.4.60. The whole sequencing procedure took about 2–3 working days.

Reads were analyzed using the “VirMet” in-house bioinformatics pipeline [22]. Briefly, reads were quality-filtered and cleaned from non-viral reads by aligning with STAR [23] against human, bacterial, bovine, and canine genomes. The remaining reads were aligned with BLAST [24] against an in-house viral database that contains > 46’000 complete viral sequences. The BLAST hit with the lowest E-value was reported, given identity was higher than 75%. Sequencing and bioinformatic analysis was done in duplicates for each sample and the results merged for further analysis. Viral reads and undetermined reads of each sample were uploaded to Zenodo (http://doi.org/10.5281/zenodo.400950).

For confirmation of identified virus hits, *de-novo* contigs were assembled with *velvet* (minimum contig length 200) [25] using only the virus specific reads reported by the VirMet pipeline and analyzed by BLAST. Enteroviruses were genotyped with an automated genotyping tool [26].

### Confirmation of Rhinovirus, TTV and HHV-7 infection by specific PCR

Rhinovirus, TTV and HHV-7 were detected with specific real-time PCR as described [27–29]. For TTV and HHV-7, copies/μl were calculated using plasmid-based standards with known concentrations in every run.

## Results

### Respiratory symptoms with etiology revealed by routine testing

**Fig 1:**
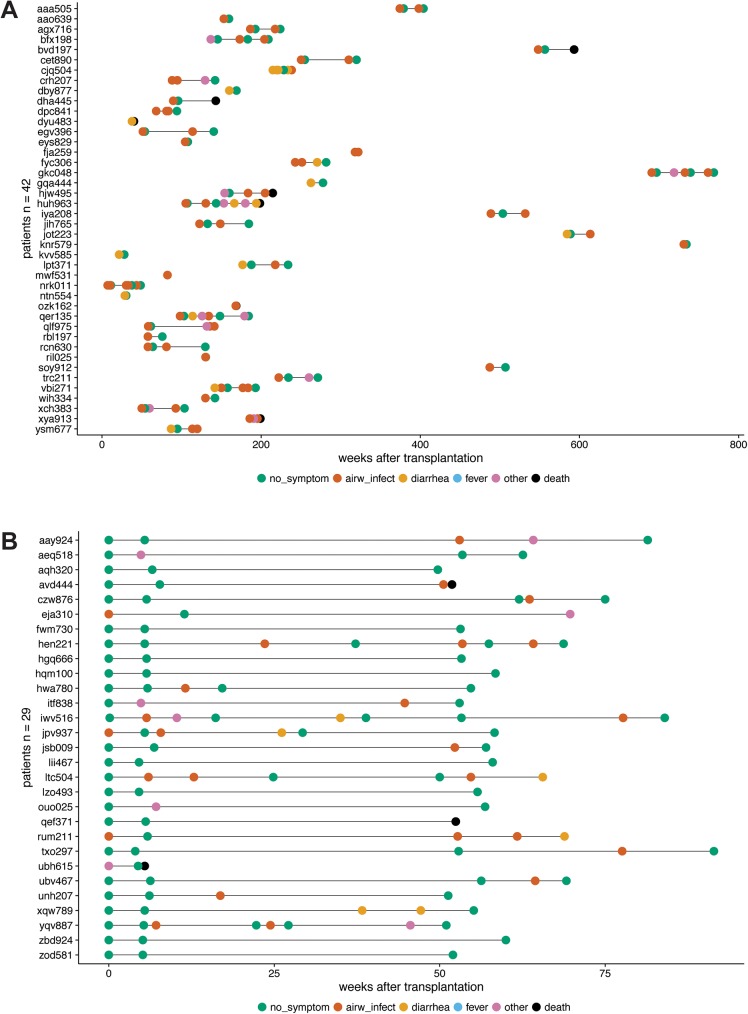
Overview of visits of lung transplant cohort. Relative timeline of visits for patients of the indication enrollment (A) and for patients of the baseline enrollment starting at the transplantation (B). Symptoms are color coded as follows: no symptoms in green, airway infection in red, diarrhea in yellow, fever in blue, other symptoms (including dyspnea and decrease in FEV1) in purple, death of the patient in black.

**Fig 2:**
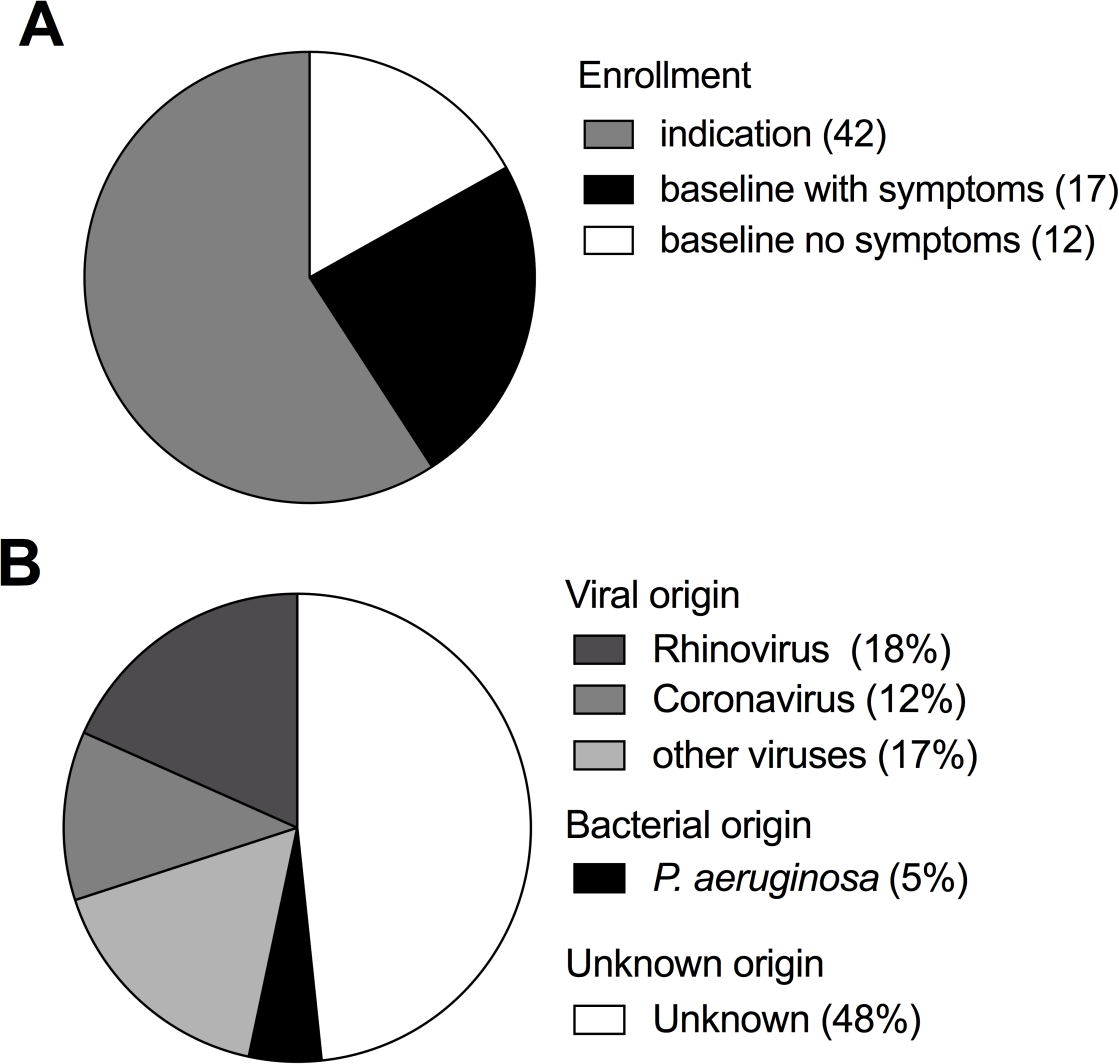
Etiology of respiratory symptoms in lung transplant patients remained undetermined in almost half of analyzed episodes. A) Number of lung transplant patients enrolled at baseline and by indication (in total 71). Patients with baseline enrollment are grouped into those that never developed symptoms and those that developed at least one symptom (airway infection or dyspnea or decrease in FEV1) during the first 15 months after transplantation. B) Etiology of respiratory symptoms among 60 observed episodes as determined by routine diagnostic testing. Episodes for which all routine diagnostic tests were negative were summarized as unknown etiology.

A total of 71 lung transplant recipients participated in this study (Table 1). Among the 29 patients enrolled at baseline, 17 showed no respiratory symptoms up to 15 months after lung transplantation whereas 12 patients developed respiratory symptoms. Among those and the additional 42 patients of the “indication enrollment”, we observed a total of 60 episodes of respiratory symptoms (Figs 1 and 2A). A viral and bacterial etiology was established by routine testing in 28 (47%) and 3 (5%) episodes, respectively. Of note, in 29 (48%) episodes, no microbial or viral etiology of infection could be determined by routine diagnostics (Fig 2B). CMV and EBV viral loads were tested frequently in blood and were negative during each of the investigated 60 respiratory episodes.

**Table 1:** Demographics of lung transplant recipients and controls.

		Lung transplant recipients	Healthy controls
**Total** (N)		71	42
**Male** (N, %)		35 (49%)	16 (38%)
**Ethnicity** (N, %)			
	White	70 (98.6%)	40 (95%)
	Asian	1 (1.4%)	2 (4.8%)
**Age** (median years, interquartile range)		50 (33–58)	38 (29–56)
**Underlying disease** (N, %)			
	Cystic fibrosis	26 (36.6%)	
	COPD^a^	19 (26.8%)	
	Interstitial lung disease	12 (16.9%)	
	Alpha 1-antitrypsin-deficiency	3 (4.2%)	
	Sarcoidosis	3 (4.2%)	
	Other	8 (11.3%)	
**Co-morbidities** (N, %)			
	Asthma		4 (9.5%)
	Rhinitis		7 (16.8%)
	Conjunctivitis		1 (4.2%)
**Enrollment scenario** (N, %)			
	Baseline enrollment	29 (40.9%)	
	Indication enrollment	42 (59.1%)	

^a^Chronic obstructive pulmonary disease

### Metagenomic sequencing of patients with respiratory symptoms of unknown etiology

We were particularly interested in the 29 episodes of respiratory infections (from 24 different patients) that were considered of unknown etiology, despite typical symptoms. We sequenced 27 throat swabs and 2 BAL samples using high-throughput metagenomic analysis (Table 2, S1 and S2 Tables) and identified reads of common respiratory viruses like Rhinovirus A and B (HRV-A/B) and Human coronavirus (CoV) in five samples. To confirm the correct identification of these viruses by our metagenomic pipeline, we performed *de-novo* assembly of all specific virus reads and used BLAST for aligning the resulting contigs against the full NCBI database. For all four cases of rhinovirus detection, between 2 and 11 contigs were assembled that were genotyped as expected to the corresponding Human rhinovirus A and B, respectively. For fyc306, de-novo alignment was not done because of the small number of reads. Direct BLAST analysis of those 12 reads produced matches to Human coronavirus genomes.

**Table 2:** Virus reads reported by the bioinformatic pipeline.

	Metagenomic Sequencing (reads)						specific real-time PCR (ct)		
ID	HRV-A	HRV-B	HRV-C	CoV	TTV	HHV-7	HRV	TTV	HHV-7
aaa505^a^					5’380		nd^b^	17.12	neg^c^
agx716		3			27’266	27	nd	22.1	41.1
bvd197						8	nd	neg	39.4
cjq504	1	1	3			318	nd	neg	29.3
dha445^a^							nd	36.3	40.2
fja259 (1/15)						132	nd	35.4	36.4
fja259 (2/15)						3	nd	37.8	39.3
fyc306				**12**		3	nd	31.7	36.5
gkc048	3	**1'431**				16	28	34.1	35.2
hjw495	1	1			1	25	nd	30.9	32.5
huh963						8	nd	29.9	33
hwa780		3			2'502		nd	23.4	neg
iwv516	8	2					nd	29.8	neg
jih765	3					5	nd	38.5	36.4
lpt371	**875**					5	22.4	neg	39.4
nrk011 (06/14)					9	2	nd	24.8	34.7
nrk011 (09/14)	**44**				56		neg	25.1	39.5
ozk162		2					nd	neg	neg
rcn630	**56**				72	2	35.9	27.6	neg
soy912						1	nd	35.2	37.5
ubh615							nd	36.8	neg
vbi271							nd	37.3	35.4
xch383 (3/14)						65	nd	35.3	34.3
xch383 (4/14)					54		nd	28.3	44.4
xya913 (02/14)						2	nd	36.0	37.8
xya913 (03/14)							nd	41.7	neg
xya913 (04/14)							nd	37.7	neg
yqv887					7	30	nd	28.9	33.6
ysm677							nd	33.4	38.4

^a^ BAL samples

^b^ not done

^c^ negative (ct > 45)

**Fig 3:**
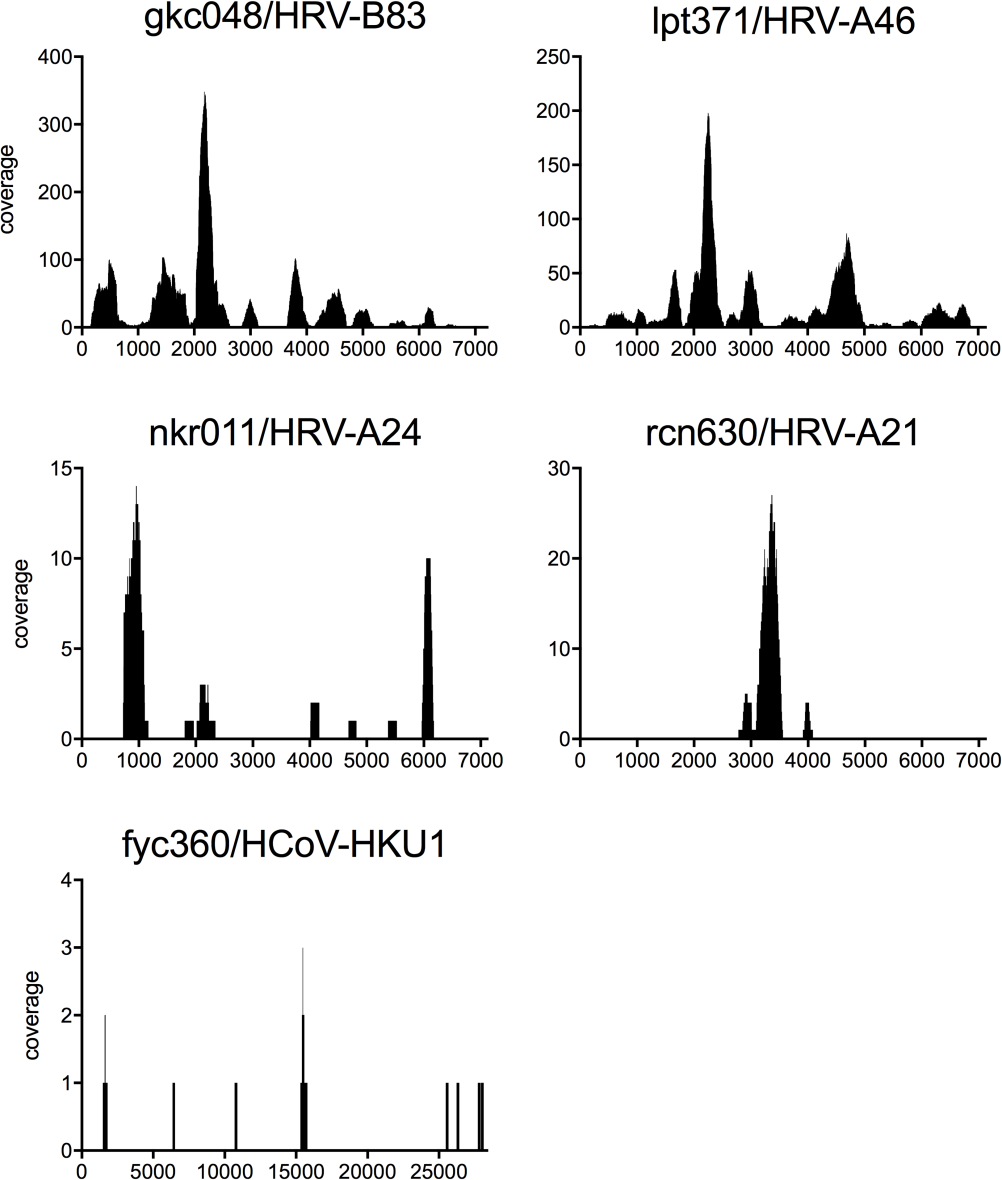
Virus reads identified were distributed over the whole reference genomes. Coverage plots for respiratory viruses identified in patients gkc048, nrk011 (09/14), lpt371, rcn630, fyc360. The coverage calculated with *samtools depth* is shown at every position on the reference genome. The following reference genomes were used for alignment: Human rhinovirus B83 strain ATCC VR-1193 (FJ445161.1) for gkc048, Human rhinovirus A46 (DQ473506.1) for lpt371, Human rhinovirus A21 strain ATCC VR-1131 (FJ445121.1) for rcn630, Human rhinovirus A24 (EF173416.1) for nrk011 (09/14), Human coronavirus HKU1 strain N5P8 (DQ339101.1) for fyc360.

Next, the most frequently recorded reference sequence reported by the pipeline was used to analyze the genome coverage. Although there were some prominent spikes in coverage, in most of the samples, the reads were distributed over several locations of the genome, giving confidence that the reads originate from sequencing a full-length virus and not from a contamination or misalignment (Fig 3). This was especially important to verify in the sample of patient fyc306, which only accounted for 12 CoV reads.

### Confirmation of respiratory virus infection by specific PCR

We used specific PCR to confirm the results for the five cases where rhinovirus and coronavirus infections were detected by metagenomic analysis. As all five patients were previously checked by multiplex PCR for the presence of respiratory viruses during routine testing but were diagnosed as negative, we first re-examined the original diagnostic test results. In two cases (gkc048 and lpt371), late amplification in the multiplex PCR was evident which, however, did not fulfill the criteria for positivity (e.g. reaching a plateau until cycle 45) and therefore was reported as negative. For patient fyc306, where CoV was detected by metagenomic sequencing, no respiratory virus diagnostics had been performed for the analyzed symptom visit. A test conducted 14 days prior to the symptom visit, however, showed evidence of late CoV amplification, but again did not fulfill the criteria for positivity. Re-analysis of the sample from the symptom visit confirmed the results of the metagenomics analysis and showed a clear positive for CoV HKU (ct 27.3).

We next conducted specific PCR for rhinovirus using a simplex assay which confirmed high viral load rhinovirus infection of patient gkc048 (ct value 28) and patient lpt371 (ct value 22). The investigated sample from patient rcn630 showed a low Rhinovirus infection (ct value 36; 52 reads detected by sequencing). In contrast rhinovirus could not be detected by PCR in patient nrk011 (09/14) (44 reads detected, Table 2).

### Frequent detection of non-respiratory viruses and bacteriophages

In addition to the common respiratory viruses reported above, we frequently identified reads for Human herpes virus 7 (HHV-7) in 18 and Torque teno virus (TTV) in 9 of the 29 samples, respectively (Table 2). TTV reads were analyzed in depth for different types and a cross contamination of samples bvd197, cjq504, fja259 (1/15) and fyc306 with TTV sequences of sample agx716 that was sequenced in the same run was detected (S3 Table). We further identified reads for other human viruses including KI polyomavirus, Human papillomavirus and Human herpesvirus 6A and 6B in individual samples (S1 Table). Reads from human endogenous retrovirus can be explained by an incomplete filtering of human reads. Our metagenomic pipeline further reported numerous reads of bacteriophages (S1 Table). All bacteriophages identified primarily infect bacteria known to colonize the upper respiratory tract [30].

### Dynamics of TTV and HHV-7 viral load in immunosuppressed patients

TTV and/or HHV-7 reads proved to be prevalent in many samples. While this was expected for TTV, an ubiquitous annellovirus known to expand under immunosuppression [31–34], the high prevalence of HHV-7 in respiratory tract samples was surprising. Specific real-time PCR confirmed all cases with HHV-7 and TTV identified by metagenomic sequencing and also identified additional cases that were not picked up in the metagenomic analysis (Table 2). Viral load levels for HHV-7 were low across all samples limiting the chances for detection by sequencing.

**Fig 4:**
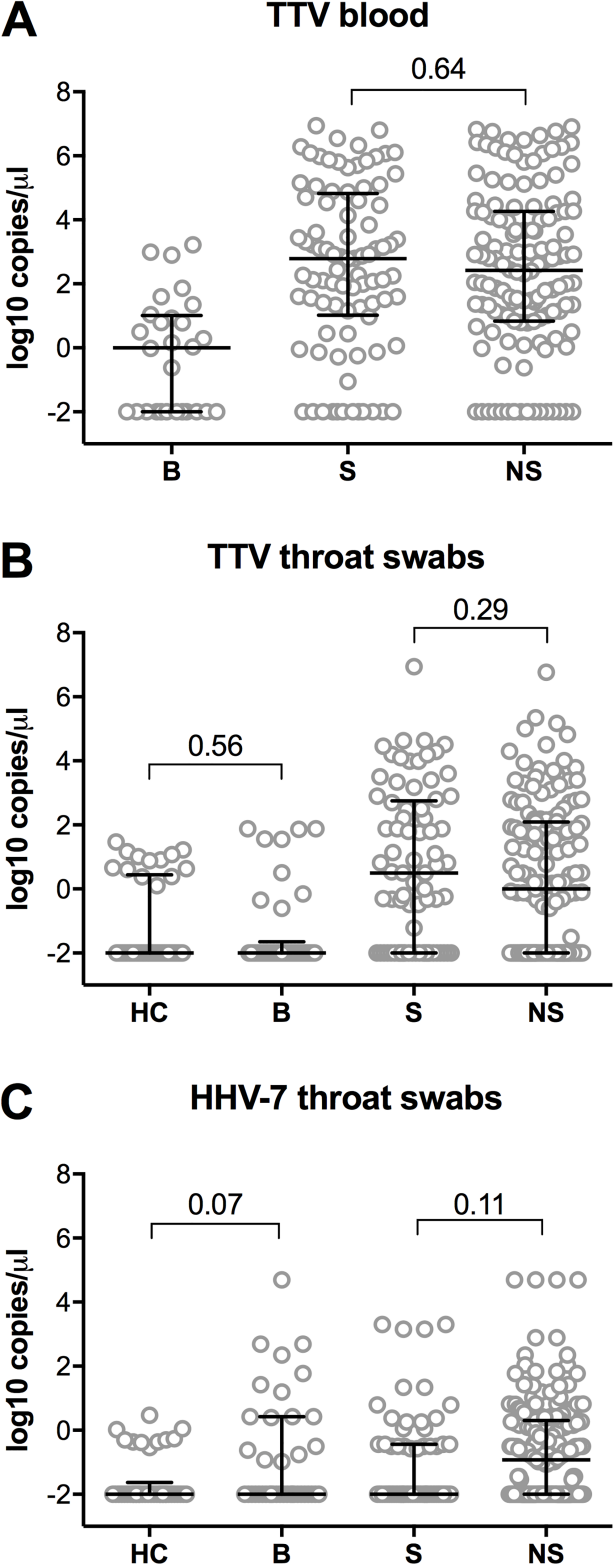
TTV and HHV-7 viral loads did not correlate with symptoms. TTV virus loads in blood samples (A), TTV viral loads in throat swabs (B) and HHV-7 viral loads in throat swabs (C). HC: healthy controls, B: baseline (time of transplantation) of baseline enrollment patients, S: symptomatic visits of both enrollment scenarios, NS: non-symptomatic visits of both enrollment scenarios. Undetermined values were set to -2 log10 copies/μl for plotting and statistical analysis. Error bars show median and interquartile ranges, *p*-values are calculated with the Mann-Whitney U test.

To assess whether the presence of these viruses was linked with either immunosuppression or respiratory infection, we measured TTV and HHV-7 levels in all available throat swabs and blood samples of the study participants and in throat swabs of healthy controls. Interestingly, HHV-7 was only detected in throat swabs and could not be detected in blood, suggesting a localized reactivation of HHV-7. Viral loads of TTV and HHV-7 proved similar amongst healthy controls and lung transplant recipients at baseline (Fig 4), showing that lung transplant recipients did not have higher viral loads at the time of transplant. Similarly, we did not see a difference in TTV and HHV-7 virus loads between symptomatic visits and symptom-free visits of both enrollment scenarios (Fig 4).

**Fig 5:**
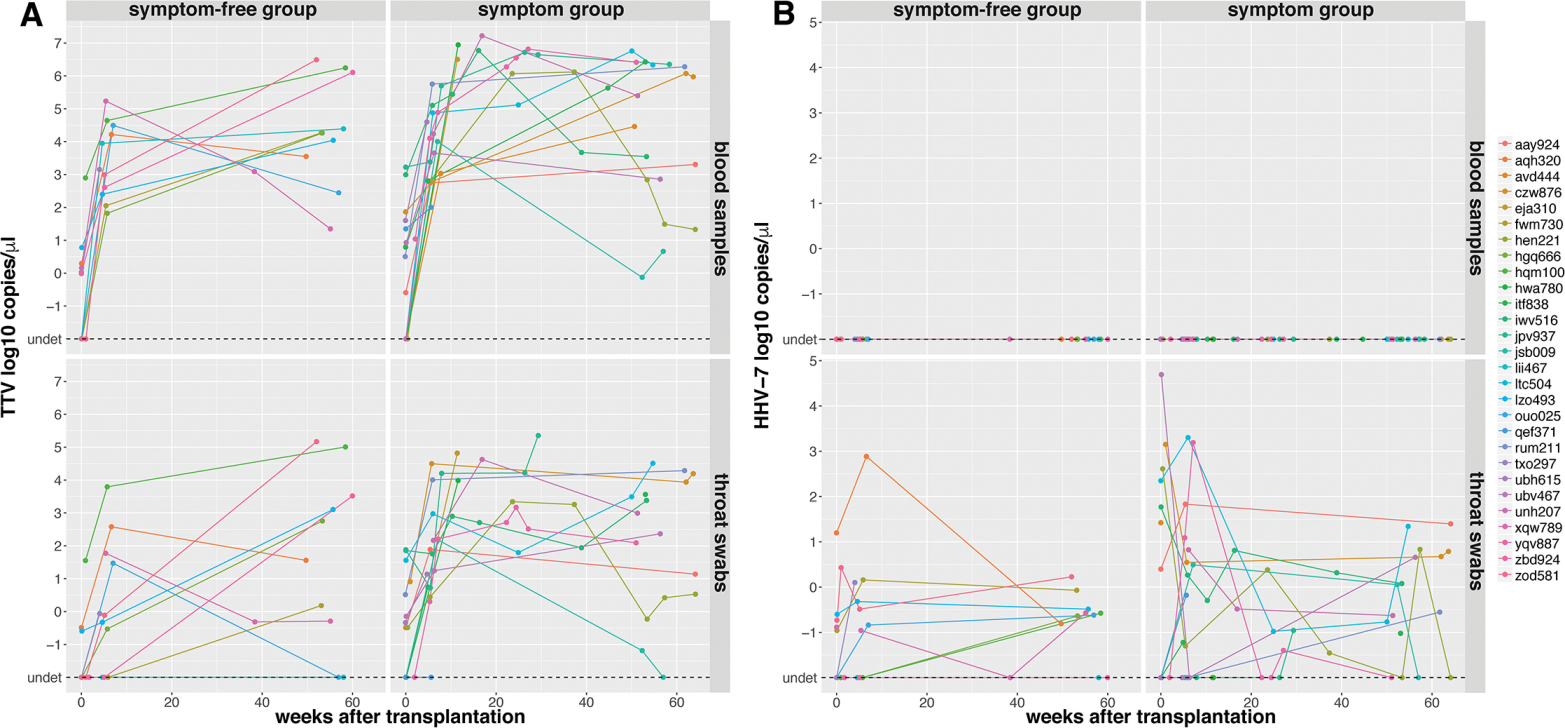
TTV virus loads increase under immunosuppressive therapy in the early post transplantation phase. A) TTV virus loads monitored longitudinally in blood and throat swab samples. Patients with baseline enrollment are grouped into those that never developed symptoms and those that developed at least one symptom (airway infection or dyspnea or decrease in FEV1) during the first 15 months after transplantation. There was no significant difference comparing the two patient groups at time of transplantation, at the visit closest to 5 weeks and at the visit closest to one year after transplantation (Kruskal-Wallis test, data not shown). Undetermined values were set to -2 log10 copies/μl for plotting and statistical analysis. B) Same as in A, but for HHV-7 virus loads.

We next stratified our patients in two groups, based on whether patients developed respiratory symptoms up to 15 months after transplantation or not and monitored the viral loads of TTV and HHV-7 longitudinally in these two patient groups. As shown in previous studies [35], TTV viral loads in swab and blood significantly increased under immunosuppression and plateaued after the first routine visit in both patient groups studied (Fig 5A). Importantly though, we observed no significant difference in viral load between symptom-free and symptomatic groups at baseline and at the visits closest to 5 weeks and to one year, respectively. HHV-7 did not follow this pattern and viral loads remained at low levels in swabs throughout the observation period and were undetectable in blood (Fig 5B).

## Discussion

In the present study we used metagenomic virus sequencing to re-analyze episodes suggestive of airway infection in lung transplant recipients that remained negative after extensive routine testing to to identify a viral infectious etiology. Focusing on viruses, our open metagenomic approach proved successful in identifying different pathogens. In five of the 29 samples, we successfully detected sequencing reads of respiratory viruses that were not accounted for in prior routine testing. Rhinovirus-specific simplex assays confirmed the presence of rhinovirus in three out of four samples with previously negative results in routine multiplex diagnostics, after HRV was detected by the metagenomic approach.

The sequencing reads were distributed over the whole genome of the respective viruses (Fig 2) highlighting that indeed a full-length genome was detected. Despite the fact that three of five detected viruses seemed of low level infection (indicated by low read numbers and high ct value), the identified respiratory viruses are certainly clinically relevant as the infections occurred in patients suffering from respiratory symptoms where despite extensive routine testing no other etiology was found.

Importantly, the metagenomic analysis detected viral pathogens in samples in which the prior routine multiplex PCR test was negative, the later confirmatory simplex PCR though positive. This was likely due to a low specificity of the PCR for the given virus strains which can occur particularly with multiplex assays [22]. However, as the primer and probe sequences from the multiplex PCR assay are not available, this is difficult to asses.

While thus metagenomic sequencing in certain cases can be more effective than routine multiplex PCR, a general drawback of metagenomic analysis remains its relatively low sensitivity compared to specific simplex PCR [36]. Indeed, the real time PCR detected TTV virus in 16 additional samples compared to metagenomic sequencing. On the other hand, detection by metagenomic techniques cannot always be correlated with virus abundance.

A number of factors influence the amount of virus in a given sample: location, technique of sampling, and pre-analytical sample preparation have a great impact on the relative amount of viral genome available to be sequenced [37]. Enrichment of viral nucleic acids from clinical samples prior to sequencing as we successfully imply in our protocol is thus crucial [38].

Metagenomic sequencing frequently detected TTV and monitoring the longitudinal dynamics of TTV by PCR showed an increase of the TTV after initiation of immunosuppression as previously described [31, 32, 35]. Nevertheless, the presence of the virus could not be linked to any relevant, clinical manifestation so far.

Most intriguingly, we identified HHV-7 in the majority of swab samples. Unlike TTV, HHV-7 showed no dynamics but remained at low levels even after initiation of immunosuppressive therapy. Interestingly, HHV-7 was restricted to the respiratory tract and not detectable in blood. The local presence of HHV-7 is intriguing and needs to be investigated in more detail to understand its significance in lung transplant recipients. Owing to its commonly low pathogenicity, clinical manifestations of HHV-7 disease are poorly described. Seroprevalence of HHV-7 in the human population is estimated to be at 90% and the primary infection with this virus typically occurs in the first 5 years of life [39]. HHV-7 has been linked to roseola-like disease, fever or CMV disease [40, 41] and secondary reactivation of the latent reservoir of this virus can take place in solid organ transplant patients in the first weeks of immunosuppressive therapy [42], frequently in combination with detection of CMV [43–45].

Data from our cohort suggest that HHV-7 replication in the respiratory tract does not depend on CMV replication. All our patients with either intermediate or high-risk constellation received CMV antiviral prophylaxis with valganciclovir and accordingly CMV was not detected in the 29 samples that were analyzed by metagenomic sequencing. Whether this medication has any influence on HHV-7 replication remains unknown [46].

While our metagenomic approach identified respiratory viruses in five of 29 samples with unknown etiology, in the remaining 24 symptomatic lung transplant samples no potentially disease related viruses were found, leaving the exact etiology of symptoms unclear. Whether this was due to insufficient sensitivity of the metagenomic approach, a novel virus not detected in the bioinformatic analysis or an etiology other than of viral origin remains unknown.

For the time being, the relevance of metagenomic findings for the treating physician still needs to be critically evaluated. A reliable metagenomics analysis must provide convincing evidence that the identified virus reads indeed indicate presence of the postulated viruses. The number of virus-specific reads, the depth of the coverage and the percent or locations of the genome covered are main parts of this information, however, no appropriate thresholds have been established in the literature so far [47]. In our hands, >10 sequencing reads and the virus genome covered at several different locations (Fig 2) provided plausible indication that the detected reads indeed indicated the presence of the postulated viruses. Nevertheless, a careful evaluation and confirmation is necessary before treatment decisions can be solely based on NGS findings.

Overall, our study highlights the potential of metagenomic virus sequencing in complex diagnostic situations such as in immunocompromised hosts, where an open metagenomic approach is a useful addition to routine test panels. Additional efforts in validation and result interpretation are needed to fully incorporate this technology in the diagnostic repertoire.

## Supporting information

S1 Table Additional virus reads identified. (DOCX)

S2 Table Raw sequencing results. (DOCX)

S3 Table Summary of TTV types identified. (DOCX)
