# Supplementary material for: Metagenomic sequencing complements routine diagnostics in identifying viral pathogens in lung transplant recipients with unknown etiology of respiratory infection
Source: PLoS One. 2017 May 23;12(5):e0177340. doi: 10.1371/journal.pone.0177340 (PMC5441588; doi:10.1371/journal.pone.0177340)
Supplement: S1 Table — (DOCX) [file pone.0177340.s001.docx]

## **S1 Table. Additional virus reads identified.**

| **ID** | **KIPyV** | **HPV** | **HHV-6 A/B** | **HERVs** | **Bacteriophages** |
| --- | --- | --- | --- | --- | --- |
| aaa505^a^ |  |  |  | 57 |  |
| agx716 |  |  |  | 65 | 8 |
| bvd197 |  | 3 | 4 | 62 | 106 |
| cjq504 |  |  | 3 | 38 | 3097 |
| dha445^a^ |  |  |  | 40 |  |
| fja259 (01/15) | 8 | 1 | 3 | 30 | 1150 |
| fja259 (02/15) | 40 | 5 |  | 25 | 54 |
| fyc306 |  |  |  | 37 | 4 |
| gkc48 |  | 2 |  | 165 | 340 |
| hjw495 |  |  |  | 74 | 1 |
| huh963 |  |  |  | 75 | 37 |
| hwa780 |  |  |  | 16 | 16 |
| jih765 |  |  |  | 100 | 7 |
| iwv516 |  |  |  | 61 | 28 |
| lpt371 |  | 11 | 5 | 6 | 92 |
| nrk011 (06/14) |  |  |  | 107 | 88 |
| nrk011 (09/14) |  |  |  | 25 | 70 |
| ozk162 |  | 3 |  | 28 | 128 |
| rcn630 |  | 1 |  | 339 | 134 |
| soy912 |  |  |  | 96 | 39 |
| ubh615 |  |  |  | 9 | 1 |
| vbi271 |  |  |  | 4 | 7 |
| xch383 (3/14) |  |  | 1 | 31 | 8 |
| xch383 (4/14) |  |  |  | 28 | 5 |
| xya913 (02/14) |  |  |  | 46 | 49 |
| xya913 (03/14) |  |  |  | 33 | 50 |
| xya913 (0414) |  |  |  | 25 | 90 |
| yqv887 |  |  | 2 | 41 | 6 |
| ysm677 |  | 3 |  | 51 | 1 |

^a^ BAL samples

KIPyV: KI Polyomavirus

HPV: Human papillomavirus

HHV-6 A/B: Human herpesvirus 6A and 6B

HERV: Sum of Human endogenous retroviruses (strains K113, K115, HCML, HCML-ARV)

Bacteriophages: Sum of phages from *Actinomyces*, *Pseudomonas*, *E. coli*, *Enterococcus*, *Enterobacteria*, *Burkholderia*, Gut phage BED-212, *Lactobacillus*, *Lactococcus*, *Staphylococcus*, *Streptococcus*, *Streptomyces*, *Rhodococcus*, *Propionobacterium*, *Acidithiobacillus*
