# Supplementary material for: Metagenomic sequencing complements routine diagnostics in identifying viral pathogens in lung transplant recipients with unknown etiology of respiratory infection
Source: PLoS One. 2017 May 23;12(5):e0177340. doi: 10.1371/journal.pone.0177340 (PMC5441588; doi:10.1371/journal.pone.0177340)
Supplement: S2 Table — (DOCX) [file pone.0177340.s002.docx]

## **S2 Table. Raw sequencing results.**

| **ID** | **Raw**  **reads** | **Quality filtered reads** | **Quality filtered reads (%)** | **Virus**  **reads** | **Virus reads within**  **filtered reads (%)** | **Run** |
| --- | --- | --- | --- | --- | --- | --- |
| aaa505 | 1'400'851 | 384'771 | 27.47 | 5'219 | 1.356 | 7 |
| agx716 | 6'075'028 | 3'469'496 | 57.11 | 26'813 | 0.773 | 1 |
| bvd197 | 5'907'790 | 1'669'980 | 28.27 | 320 | 0.019 | 1 |
| cjq504 | 4'935'641 | 2'422'096 | 49.07 | 3'499 | 0.144 | 1 |
| dha445 | 2'386'967 | 661'756 | 27.72 | 70 | 0.011 | 7 |
| fja259 (01/15) | 7'182'842 | 4'195'776 | 58.41 | 1'415 | 0.034 | 1 |
| fja259 (02/15) | 4'284'304 | 2'704'549 | 63.13 | 196 | 0.007 | 8 |
| fyc306 | 4'325'106 | 2'257'332 | 52.19 | 68 | 0.003 | 1 |
| gkc048 | 5'748'544 | 2'114'876 | 36.79 | 1'989 | 0.094 | 2 |
| hjw495 | 6'001'302 | 1'482'285 | 24.70 | 171 | 0.012 | 2 |
| huh963 | 4'792'469 | 1'316'389 | 27.47 | 149 | 0.011 | 2 |
| hwa780 | 5'357'116 | 2'158'861 | 40.30 | 2'686 | 0.124 | 6 |
| jih765 | 4'918'574 | 3'124'706 | 63.53 | 135 | 0.004 | 2 |
| iwv516 | 5'504'126 | 1'832'396 | 33.29 | 3'139 | 0.171 | 3 |
| lpt371 | 5'950'887 | 5'088'006 | 85.50 | 1'001 | 0.020 | 3 |
| nrk011 (06/14) | 2'910'403 | 1'831'376 | 62.93 | 235 | 0.013 | 3 |
| nrk011 (09/14) | 5'884'778 | 3'700'049 | 62.87 | 232 | 0.006 | 3 |
| ozk162 | 4'341'609 | 1'925'967 | 44.36 | 210 | 0.011 | 6 |
| rcn630 | 11'395'621 | 6'443'309 | 56.54 | 627 | 0.010 | 4 |
| soy912 | 5'375'877 | 1'635'449 | 30.42 | 216 | 0.013 | 4 |
| ubh615 | 2'703'174 | 469'959 | 17.39 | 19 | 0.004 | 4 |
| vbi271 | 5'221'618 | 3'406'717 | 65.24 | 14 | 0.004 | 4 |
| xch383 (03/14) | 6'121'321 | 1'075'530 | 17.57 | 121 | 0.011 | 5 |
| xch383 (04/14) | 4'270'064 | 2'616'921 | 61.29 | 89 | 0.003 | 8 |
| xya913 (02/14) | 4'102'444 | 1'124'717 | 27.42 | 123 | 0.011 | 5 |
| xya913 (03/14) | 2'269'059 | 1'221'988 | 53.85 | 2'488 | 0.200 | 8 |
| xya913 (0414) | 5'276'602 | 2'208'760 | 41.86 | 396 | 0.020 | 6 |
| yqv887 | 5'159'812 | 3'794'174 | 73.53 | 103 | 0.003 | 5 |
| ysm677 | 4'962'121 | 931'971 | 18.78 | 64 | 0.007 | 5 |
