# Supplementary material for: Metagenomic sequencing complements routine diagnostics in identifying viral pathogens in lung transplant recipients with unknown etiology of respiratory infection
Source: PLoS One. 2017 May 23;12(5):e0177340. doi: 10.1371/journal.pone.0177340 (PMC5441588; doi:10.1371/journal.pone.0177340)
Supplement: S3 Table — (DOCX) [file pone.0177340.s003.docx]

## **S3 Table. Summary of TTV types identified.**

| **aaa505** |  |  |
| --- | --- | --- |
| **Reads** | **Accession** | **Virus type** |
| 5345 | KJ194503.1 | UNVERIFIED: Torque teno virus isolate TTV_Amsterdam_1994, complete genome |
| 17 | AB064607.1 | Torque teno virus 10 DNA, complete genome, isolate: JT34F |
| 12 | AF122914.3 | TT virus isolate JA20, complete genome |
| 4 | AF122913.1 | TT virus isolate GH1, complete genome |
| 1 | AF247137.1 | TT virus isolate TUPB, complete genome |
| 1 | AB064604.1 | Torque teno virus DNA, complete genome, isolate: CT39F |
|  |  |  |
| **agx716** |  |  |
| **Reads** | **Accession** | **Virus type** |
| 25419 | FR848325.1 | Torque teno virus complete genome, isolate HD13a gsB20.33 |
| 905 | FR848327.1 | Torque teno virus complete genome, isolate HD13c gsB21.51 |
| 349 | FR848326.1 | Torque teno virus complete genome, isolate HD13b gsB20.58 |
| 336 | AB017613.1 | Torque teno virus 16 DNA, complete genome, isolate: TUS01 |
| 77 | KJ082064.1 | Torque teno virus isolate TTV-Hebei-1, complete genome |
| 30 | AB054647.1 | Torque teno virus 8 DNA, complete genome, genotype 22 |
| 27 | AB064607.1 | Torque teno virus 10 DNA, complete genome, isolate: JT34F |
| 21 | AF261761.1 | Torque teno virus 7 isolate PMV, complete genome |
| 21 | FR751492.1 | Torque teno virus complete genome, isolate TTV-HD20a (uro960) |
| 20 | AJ620219.1 | Torque teno virus, isolate tth9, complete genome |
| 12 | FR751495.1 | Torque teno virus complete genome, isolate TTV-HD20d (uro746) |
| 9 | AB025946.2 | Torque teno virus 19 DNA, complete genome, isolate: TTV SANBAN |
| 6 | FR751493.1 | Torque teno virus complete genome, isolate TTV-HD20b (uro742) |
| 6 | AY666122.2 | Torque teno virus 3 strain HEL32, complete genome |
| 5 | AB064605.1 | Torque teno virus 12 DNA, complete genome, isolate: CT44F |
| 4 | GU797360.1 | Torque teno virus 10 isolate BIS8-17, complete genome |
| 4 | AF435014.1 | Torque teno virus 6 isolate KAV, complete genome |
| 4 | AF298585.1 | TT virus Polish isolate P/1C1, complete genome |
| 3 | AF122913.1 | TT virus isolate GH1, complete genome |
| 3 | AB054648.1 | Torque teno virus DNA, complete genome, genotype 23 |
| 2 | JN980171.1 | Torque teno virus isolate TPK01, complete genome |
| 1 | AF247137.1 | TT virus isolate TUPB, complete genome |
| 1 | AF122914.3 | TT virus isolate JA20, complete genome |
| 1 | FR751488.1 | Torque teno virus complete genome, isolate TTV-HD17 (uro702) |
|  |  |  |
| **bvd197** |  |  |
| **Reads** | **Accession** | **Virus type** |
| ~~37~~ | ~~FR848325.1~~ ^a^ | ~~Torque teno virus complete genome, isolate HD13a gsB20.33~~ |
| ~~3~~ | ~~FR848326.1~~ | ~~Torque teno virus complete genome, isolate HD13b gsB20.58~~ |
| ~~1~~ | ~~FR848327.1~~ | ~~Torque teno virus complete genome, isolate HD13c gsB21.51~~ |
|  |  |  |
| **cjq504** |  |  |
| **Reads** | **Accession** | **Virus type** |
| ~~14~~ | ~~FR848325.1~~ ^a^ | ~~Torque teno virus complete genome, isolate HD13a gsB20.33~~ |
| ~~1~~ | ~~AF298585.1~~ | ~~TT virus Polish isolate P/1C1, complete genome~~ |
|  |  |  |
| **fja259 (1/15)** |  |  |
| **Reads** | **Accession** | **Virus type** |
| ~~34~~ | ~~FR848325.1~~ ^a^ | ~~Torque teno virus complete genome, isolate HD13a gsB20.33~~ |
| ~~2~~ | ~~AB017613.1~~ | ~~Torque teno virus 16 DNA, complete genome, isolate: TUS01~~ |
|  |  |  |
| **fyc306** |  |  |
| **Reads** | **Accession** | **Virus type** |
| ~~6~~ | ~~FR848325.1~~ ^a^ | ~~Torque teno virus complete genome, isolate HD13a gsB20.33~~ |
| ~~1~~ | ~~AB017613.1~~ | ~~Torque teno virus 16 DNA, complete genome, isolate: TUS01~~ |
|  |  |  |
| **hjw495** |  |  |
| **Reads** | **Accession** | **Virus type** |
| 1 | KP036971.1 | Torque teno virus isolate TTV-2013, complete genome |
|  |  |  |
| **hwa780** |  |  |
| **Reads** | **Accession** | **Virus type** |
| 1288 | FR751490.1 | Torque teno virus complete genome, isolate TTV-HD18b (uro705) |
| 1150 | FR751489.1 | Torque teno virus complete genome, isolate TTV-HD18a (uro703) |
| 10 | AF122914.3 | TT virus isolate JA20, complete genome |
| 9 | FR751477.1 | Torque teno virus complete genome, isolate TTV-HD16c (gbCsCt43.3) |
| 6 | AF247137.1 | TT virus isolate TUPB, complete genome |
| 6 | FR751478.1 | Torque teno virus complete genome, isolate TTV-HD16b (gbCsCt43.1) |
| 5 | AY823988.1 | Torque teno virus isolate 2h, complete genome |
| 5 | FR751476.1 | Torque teno virus complete genome, isolate TTV-HD16a (gbCsCt43.2) |
| 4 | AY666122.2 | Torque teno virus 3 strain HEL32, complete genome |
| 3 | AB054648.1 | Torque teno virus DNA, complete genome, genotype 23 |
| 2 | KJ194502.1 | UNVERIFIED: Torque teno virus isolate TTV_Amsterdam_1995, complete genome |
| 2 | AY823989.1 | Torque teno virus isolate 3h, complete genome |
| 2 | FR751492.1 | Torque teno virus complete genome, isolate TTV-HD20a (uro960) |
| 2 | AF435014.1 | Torque teno virus 6 isolate KAV, complete genome |
| 2 | AB054647.1 | Torque teno virus 8 DNA, complete genome, genotype 22 |
| 1 | KP343841.1 | UNVERIFIED: Torque teno virus isolate S74, complete genome |
| 1 | AB025946.2 | Torque teno virus 19 DNA, complete genome, isolate: TTV SANBAN |
| 1 | AJ620227.1 | Torque teno virus, isolate tth5, complete genome |
| 1 | AJ620226.1 | Torque teno virus, isolate tth4, complete genome |
| 1 | AF122913.1 | TT virus isolate GH1, complete genome |
| 1 | FR751485.1 | Torque teno virus complete genome, isolate TTV-HD16j (gbDhDi43.6) |
|  |  |  |
| **nrk011 (06/14)** |  |  |
| **Reads** | **Accession** | **Virus type** |
| 3 | AF247137.1 | TT virus isolate TUPB, complete genome |
| 2 | AB028668.1 | Torque teno virus 15 DNA, complete genome, isolate: TJN01 |
| 2 | AB017613.1 | Torque teno virus 16 DNA, complete genome, isolate: TUS01 |
| 1 | KJ194619.1 | Rodent Torque teno virus 1 isolate AS_WM1_Se_2, complete genome |
| 1 | AJ620218.1 | Torque teno virus, isolate tth3, complete genome |
|  |  |  |
| **nrk011 (09/14)** |  |  |
| **Reads** | **Accession** | **Virus type** |
| 19 | AB028668.1 | Torque teno virus 15 DNA, complete genome, isolate: TJN01 |
| 19 | AB017613.1 | Torque teno virus 16 DNA, complete genome, isolate: TUS01 |
| 8 | AF247137.1 | TT virus isolate TUPB, complete genome |
| 4 | AB054647.1 | Torque teno virus 8 DNA, complete genome, genotype 22 |
| 2 | AY823989.1 | Torque teno virus isolate 3h, complete genome |
| 2 | FR751490.1 | Torque teno virus complete genome, isolate TTV-HD18b (uro705) |
| 1 | FR751489.1 | Torque teno virus complete genome, isolate TTV-HD18a (uro703) |
| 1 | FR751477.1 | Torque teno virus complete genome, isolate TTV-HD16c (gbCsCt43.3) |
|  |  |  |
| **rcn630** |  |  |
| **Reads** | **Accession** | **Virus type** |
| 24 | AB025946.2 | Torque teno virus 19 DNA, complete genome, isolate: TTV SANBAN |
| 14 | AB054647.1 | Torque teno virus 8 DNA, complete genome, genotype 22 |
| 7 | FR751481.1 | Torque teno virus complete genome, isolate TTV-HD16f (gbCuCv43.2) |
| 7 | FR751477.1 | Torque teno virus complete genome, isolate TTV-HD16c (gbCsCt43.3) |
| 5 | FR751492.1 | Torque teno virus complete genome, isolate TTV-HD20a (uro960) |
| 4 | AY823989.1 | Torque teno virus isolate 3h, complete genome |
| 4 | FR751476.1 | Torque teno virus complete genome, isolate TTV-HD16a (gbCsCt43.2) |
| 2 | AF247137.1 | TT virus isolate TUPB, complete genome |
| 2 | FR751498.1 | Torque teno virus complete genome, isolate TTV-HD21 (rheu111) |
| 1 | AF122914.3 | TT virus isolate JA20, complete genome |
| 1 | AB017610.1 | TT virus genotype 1a DNA, complete genome |
| 1 | FR751490.1 | Torque teno virus complete genome, isolate TTV-HD18b (uro705) |
|  |  |  |
| **xch383 (4/14)** |  |  |
| **Reads** | **Accession** | **Virus type** |
| 29 | FR751492.1 | Torque teno virus complete genome, isolate TTV-HD20a (uro960) |
| 17 | KP343840.1 | UNVERIFIED: Torque teno virus isolate S73, complete genome |
| 3 | FR751489.1 | Torque teno virus complete genome, isolate TTV-HD18a (uro703) |
| 2 | AY823988.1 | Torque teno virus isolate 2h, complete genome |
| 1 | KJ194502.1 | UNVERIFIED: Torque teno virus isolate TTV_Amsterdam_1995, complete genome |
| 1 | KJ082064.1 | Torque teno virus isolate TTV-Hebei-1, complete genome |
| 1 | FR751498.1 | Torque teno virus complete genome, isolate TTV-HD21 (rheu111) |
|  |  |  |
| **yqv887** |  |  |
| **Reads** | **Accession** | **Virus type** |
| 6 | FR751489.1 | Torque teno virus complete genome, isolate TTV-HD18a (uro703) |
| 1 | AF247137.1 | TT virus isolate TUPB, complete genome |

^a^Strikethrough reads were considered contamination.
